# Role of Uremic Toxins in Vascular Inflammation Associated with Chronic Kidney Disease

**DOI:** 10.3390/jcm13237149

**Published:** 2024-11-26

**Authors:** Rania Chermiti, Stéphane Burtey, Laetitia Dou

**Affiliations:** 1 C2VN, Aix-Marseille University, INSERM, INRAE, 13005 Marseille, France; raniachermiti123@gmail.com (R.C.); stephane.burtey@univ-amu.fr (S.B.); 2 Centre de Néphrologie et Transplantation Rénale, APHM, Hôpital Conception, 13005 Marseille, France

**Keywords:** chronic kidney disease, vascular inflammation, cardiovascular disease, uremic toxins, aryl hydrocarbon receptor

## Abstract

Cardiovascular disease (CVD) is a major complication of chronic kidney disease (CKD), despite improvements in patient care. Vascular inflammation is a crucial process in the pathogenesis of CVD and a critical factor in the cardiovascular complications in CKD patients. CKD promotes a pro-inflammatory environment that impacts the vascular wall, leading to endothelial dysfunction, increased oxidative stress, and vascular remodeling. The uremic toxins that accumulate as kidney function declines are key contributors to vascular inflammatory processes. Our review will examine how CKD leads to vascular inflammation, paving the way to CVD. We will provide an overview of the mechanisms of vascular inflammation induced by uremic toxins, with a particular focus on those derived from tryptophan metabolism. These toxins, along with their receptor, the aryl hydrocarbon receptor (AHR), have emerged as key players linking inflammation and thrombosis. A deeper understanding of the mechanisms underlying inflammation in CKD, particularly those driven by uremic toxins, could reveal valuable therapeutic targets to alleviate the burden of CVD in CKD patients.

## 1. Introduction

Chronic kidney disease (CKD) is a complex condition characterized by the gradual loss of kidney function over time. CKD is associated with inflammation in the kidney and with low-grade systemic inflammation primarily affecting the cardiovascular system [1]. This systemic inflammation is revealed in patients with CKD by increased levels of the inflammatory markers high-sensitivity C-reactive protein (hsCRP) and fibrinogen as well as pro-inflammatory cytokines such as interleukin-1 (IL-1), interleukin-6 (IL-6), and tumor necrosis factor-alpha (TNFα), which correlate with the decrease in kidney function [2].

Inflammation plays a significant role in the progression of CKD, and there are several mediators involved in this process. Inflammatory immune cells, particularly macrophages, infiltrate the kidneys during CKD and release inflammatory cytokines and enzymes that contribute to tissue damage and favor kidney fibrosis [3,4,5]. Chemokines such as monocyte chemoattractant protein-1 (MCP-1) play a role in recruiting inflammatory cells to the kidneys [3]. Endothelium dysfunction promotes immune cell infiltration into the kidney through increased expression of adhesion molecules and contributes to the inflammatory processes that lead to renal fibrosis [6,7]. Increased oxidative stress and the production of reactive oxygen species can damage kidney cells and promote inflammation [8]. An upregulation of NLRP3 inflammasome, a multiprotein complex involved in the production of pro-inflammatory cytokines, was shown in the kidneys of patients with CKD and contributes to kidney injury in mice [9]. The family of pro-inflammatory transcription factors nuclear factor-κB (NF-κB) can be activated in kidney cells by a variety of stimuli found in CKD, including oxidative stress, pro-inflammatory cytokines, angiotensin II, activation of the NLRP3 inflammasome, or uremic toxins [5,10]. In turn, NF-κB activation results in the production of pro-inflammatory mediators, forming a positive feedback loop that sustains the inflammatory response [5,10]. Other pathways leading to chronic inflammation contribute to the development of CKD, such as dysregulation of the complement system or activation of Toll-like receptors (TLRs) by endogenous molecules released during tissue damage such as damage-associated molecular patterns (DAMPs) [5].

Progression of CKD leads to an increase in the inflammatory load, aggravating the damage to the kidney and the cardiovascular system. CKD increases the risk and severity of all forms of CVD: coronary heart disease, heart failure, atrial fibrillation, peripheral artery disease, and venous thromboembolism [11]. Traditional risk factors of CVD, like diabetes, hypertension, and tobacco use, are more frequent in patients with CKD; however, they do not fully account for the high prevalence of CVD, which remains the leading cause of mortality and morbidity in CKD [12]. Consequently, nontraditional risk factors, including inflammation, uremic toxins, vascular calcifications, and endothelial dysfunction, have been recognized as important contributors to the development and severity of CVD in CKD patients [13]. The causal link between inflammation and CVD was provided by the results of many clinical trials using anti-inflammatory drugs to reduce the cardiovascular risk [14,15]. In patients with CKD, inflammation assessed by hs-CRP or IL-6 significantly predicts major cardiovascular events and could explain the residual cardiovascular risk despite statin therapy [16].

## 2. Vascular Inflammation in CKD

CKD causes structural changes and vascular dysfunction, affecting both macro- and microvessels [7,17]. It induces arterial stiffness with increased wall thickening, vascular calcifications linked to calcium deposition in the arterial wall, intimal hyperplasia, and accelerated atherosclerosis [17,18].

The arteries of patients with CKD show a pro-inflammatory, pro-oxidant, pro-aggregating, and vasoconstrictive profile with a macrophagic infiltrate [19,20]. A microarray study showed that most of the deregulated mRNAs are associated with cell signaling, including increased pro-inflammatory TNFα/NF-κB signaling, decreased TGF-β signaling, and increased pathways related to protein degradation by ubiquitination [19]. Arteries from patients with CKD show a dysregulation of inflammation linked to the prostaglandin pathway, manifested by an enhanced expression of COX-2/cPLA-2 [20], and a significant decrease in the expression of prostacyclin synthase (PTGIS) [19], the membrane enzyme producing the anti-inflammatory prostaglandin, prostacyclin (PGI2). Furthermore, the pro-inflammatory profile of CKD arteries is revealed by the activation of inflammasome, with an increased tissue expression of IL-1β, caspase-1, and NLRP3 [20].

Vascular inflammation in CKD contributes to interconnected processes that exacerbate one another in a vicious circle, leading to structural changes in blood vessels, including thickening of the vessel walls and remodeling of the arterial architecture. These changes result in reduced blood flow and increased vascular stiffness. CKD induces endothelium dysfunction and increased oxidative stress, due to both excessive production of reactive oxygen (ROS) and reactive nitrogen (RNS) species and antioxidant depletion [21]. Oxidative stress impairs nitric oxide (NO) bioavailability [21], induces lipid oxidation, and contributes to inflammation [22]. Inflammation and oxidative stress damage the endothelium and induce the dysfunction of endothelial and vascular smooth muscle cells, leading to reduced production of NO and impaired vasodilation [23]. In turn, endothelial dysfunction participates in inflammation and contributes to vasoconstriction, hypertension [24], and vascular remodeling [25]. Inflammatory cells can infiltrate the blood vessel wall in response to signals from injured tissues and circulating inflammatory factors, and they release pro-inflammatory cytokines, chemokines, and enzymes that contribute to inflammation and tissue damage [26]. The increase in levels of pro-inflammatory cytokines in CKD patients contributes to systemic inflammation and can directly impact the blood vessels [2].

Inflammation plays a significant role in the development of vascular calcifications, through the release by inflammatory cells of factors that promote the deposition of calcium and phosphate in the vessel wall, leading to vascular stiffness and reduced elasticity [27]. The release of DAMP proteins during tissue damage, along with their recognition by TLRs, is also involved in the pathophysiology of vascular calcifications in CKD, as demonstrated with calprotectin, which has been epidemiologically and mechanistically associated with vascular calcifications [28]. Hyperphosphatemia resulting from impaired phosphate excretion, along with dysregulation of calcification promoters and inhibitors, contributes to the processes linking inflammation and vascular calcification in CKD patients. In addition, phosphate levels show a positive correlation with hemoglobin levels in hemodialysis patients [29]. Phosphate may trigger inflammation partly through the induction of oxidative stress and NF-κB activation, and it induces the osteogenic differentiation of VSMCs, which may subsequently release inflammatory mediators [30]. Thus, elevated serum phosphate levels contribute not only to vascular calcification but also to inflammation and endothelial dysfunction [31,32]. The klotho/FGF23 axis, the principal regulator of phosphate homeostasis, is also dysregulated in CKD patients. Klotho expression is down-regulated in CKD due to systemic or renal inflammation, notably through inflammatory cytokines activating NF-κB [33]. Klotho is a potent inhibitor of vascular calcifications [30] that enhances vasorelaxation [34]. It attenuates endothelial inflammation by inhibiting NF-κB nuclear translocation and reducing oxidative stress, increases endothelial NO production [34], and protects endothelial cells from senescence induced by the uremic environment [30,35]. The decreased expression of klotho in CKD is probably a key player in vascular dysfunction, as klotho deficiency increases vascular calcification in mice [34] and is related to enhanced oxidative stress in hemodialysis patients [36]. FGF-23, on the other hand, is elevated in CKD and correlates with dialysis vintage in patients undergoing dialysis [37]. Its levels are also influenced by the dialysis modality, as they are significantly higher in hemodialysis patients than in peritoneal dialysis patients [37]. High levels of FGF-23 change the VSMC phenotype from contractile to synthetic, increase arterial stiffening, and enhance vascular wall thickness [38]. In hemodialysis patients, elevated levels of FGF-23 are associated with inflammation, vascular calcification, and adverse cardiovascular outcomes [39]. Among pro-calcifying stimuli, osteoprotegerin, a member of the soluble receptors from the TNF superfamily that inhibits osteoclast differentiation and activation through the blockage of RANK/RANKL interaction, was proposed as an early biomarker of coronary calcification [40]. High levels of osteoprotegerin are associated with all-cause and cardiovascular mortality in patients with CKD [41]. Osteoprotegerin was recently shown to be involved in the development of vascular inflammation and endothelial dysfunction induced by angiotensin II [42]. Finally, the downregulation of fetuin-A, an antagonist of TGF-β that regulates cytokine-dependent osteogenesis, plays a role in the inflammatory mechanisms leading to vascular calcifications. Hemodialysis patients exhibit low concentrations of fetuin-A, which are associated with increased CRP and enhanced cardiovascular and all-cause mortality [43]. Fetuin-A is downregulated in inflammatory conditions and serves as a crucial inhibitor of calcification in vivo, as fetuin-A-deficient mice develop severe soft-tissue and small arterial calcifications [44].

Beyond its effects on vascular calcifications, vascular inflammation in CKD is also closely linked to increased procoagulant mediators with inflammatory properties, notably those initiated by tissue factors [45]. Such inflammatory processes can accelerate the progression of atherosclerosis, increase the risk of blood clot formation, and contribute to heart attacks, strokes, and other cardiovascular events [46].

## 3. Role of Uremic Toxins in Vascular Inflammation

Key factors in the vascular inflammation associated with CKD are uremic toxins, a diverse group of waste products that accumulate in the body when kidney function is impaired [47]. Over 100 uremic toxins have been described and classified by the European Uremic Toxin (EUTOX) Working Group [48]. Many of these toxins contribute to chronic inflammation in the context of CKD as well as to certain cardiovascular complications and dysfunction of the arteriovenous access used for hemodialysis [49]. It is important to note that the mechanisms by which these uremic toxins promote inflammation are interconnected and complex. Additionally, individual responses to these toxins can vary based on genetics, comorbidities, and other factors.

Uremic toxins with described pro-inflammatory effects include asymmetric and symmetric dimethylarginine (ADMA and SDMA), homocysteine, urea, and advanced glycation end products (AGEs); the gut-derived uremic toxins trimethylamine N-oxide (TMAO), hippuric acid, and p-cresyl sulfate; and tryptophan-derived uremic toxins like kynurenines, indoxyl sulfate, and indole-3 acetic acid (IAA).

One key characteristic of uremic toxins is that nearly all of them correlate with the extent of deterioration in kidney function, with their concentrations rising as the glomerular filtration rate declines [50]. This was notably demonstrated for ADMA, SDMA, TMAO, hippuric acid, indoxyl sulfate, IAA, and p-cresyl sulfate, whose plasma levels negatively correlated with eGFR in CKD patients [51,52,53]. Some uremic toxins are associated with CKD progression, although there is a lack of data on the relationship between their levels and the underlying kidney diseases. This is particularly true for p-cresyl sulfate and indoxyl sulfate, which both predict the progression of CKD [51]. However, the association of indoxyl sulfate with CKD progression disappears after adjustment for p-cresyl sulfate [51], whereas that of p-cresyl sulfate persists after adjustment for indoxyl sulfate [51]. In a remarkable editorial [50], Meijers and Evenepoel suggest that serum concentrations of indoxyl sulfate and p-cresyl sulfate should not be considered interchangeable predictive factors for CKD progression, and that p-cresyl sulfate may be a more reliable predictor of CKD progression than indoxyl sulfate. The example of indoxyl sulfate and p-cresyl sulfate illustrates that these uremic toxins could serve as both markers and effectors of CKD progression. While their production depends on nutrient intake, their clearance relies on specific tubular transporters, and their concentrations may reflect tubular damage [50]. Additionally, these uremic toxins may contribute to the progression of CKD by negatively affecting the renal tubular cells, notably through cytotoxic, pro-oxidant, and inflammatory mechanisms [54,55,56], and by inducing tubulointerstitial fibrosis and glomerular sclerosis [57].

One might have expected that hemodialysis, by clearing uremic toxins, would reduce inflammation in CKD patients. However, certain triggers of inflammation are directly related to hemodialysis treatment, including dialysis membranes, vascular access, and contamination of dialysis solutions [30]. In addition, whereas water-soluble toxins like TMAO are efficiently cleared by dialysis, protein-bound toxins, including AGEs, homocysteine, hippuric acid, p-cresyl sulfate, and indoxyl sulfate, are poorly cleared by dialysis due to their protein-binding property. Uremic toxins also include a number of inflammatory cytokines (IL-6, TNFα, IL-1β, IL-18) and a chemokine (IL-8), which are considered to be secondary uremic toxins with a very low dialyzability [1,58]. However, pre-dialysis levels of uremic toxins depend only very partially on their dialyzability. The levels of TMAO, which is water-soluble, along with the levels of hippuric acid, indoxyl sulfate, and IAA, which are protein-bound, are all significantly higher in hemodialysis patients than in CKD stage 5 patients [59]. In addition, the levels of ADMA are higher, whereas the levels of SDMA are lower, in hemodialysis patients compared with CKD stage 5 patients [59]. Finally, p-cresyl sulfate plasma levels do not differ between CKD stages 4–5 patients and hemodialysis patients [60]. This illustrates that pre-dialysis levels of uremic toxins are strongly influenced by numerous other factors including diet, residual renal function, the type of renal replacement therapy [61,62,63], and, particularly, inflammation (see below).

We will now examine how the elevated levels of uremic toxins in CKD contribute to vascular inflammation and explore the mechanisms through which they promote the development of CVD.

### 3.1. Inflammatory Cytokines

#### 3.1.1. Tumor Necrosis Factor-α (TNFα)

TNFα is a pro-inflammatory cytokine synthesized by activated T cells, macrophages, and mast cells, and by other cells including vascular endothelial cells, VSMCs, and cardiomyocytes. There are two main TNFα receptors (TNFRs): type 1 (TNFR1), which is ubiquitously expressed, and type 2 (TNFR2), which is found mainly in cells of the immune system. TNFα plasma levels are increased in patients with CKD, inversely correlate with GFR, and independently predict all-cause mortality in incident dialysis patients [1,58,64]. TNFα could contribute to vascular wall inflammation and atherosclerosis. By binding to membrane TNFRs and inducing downstream NF-κB and AP-1 activation, it increases the expression of the endothelial adhesion molecules VCAM-1, ICAM-1, and E-selectin and upregulates the expression of tissue factor in endothelial cells [1,65]. TNFα stimulates the release of chemokines like MCP-1 and inflammatory cytokines, including the uremic toxins IL-1β, IL-6, and IL-8, and promotes cell death [58]. TNFα present in uremic serum could participate in vascular calcifications by promoting osteoblastic transition and VSMC calcification through the upregulation of IL-6 expression via the ERK/AP-1 pathway, as evidenced in in vitro experiments with a TNFα-blocking antibody [66].

Membrane-bound, signal-transducing TNFRs can be cleaved into soluble receptors (sTNFRs) that compete with the transmembrane forms and inhibit the activity of circulating TNFα. Like TNFα, sTNFR plasma levels are elevated in CKD patients and inversely correlate with GFR [1,58]. The elevation of sTNFRs could inhibit TNFα activity by reducing the levels of free, active TNFα. However, this elevation may also indirectly indicate increased TNFα activity, as TNFα induces the release of sTNFRs into the circulation [58]. In CKD patients, the literature data suggest that serum levels of sTNFRs would rather reflect increased TNF activity, as they predict CKD progression, cardiovascular events, and mortality in dialysis patients [58]. Additionally, sTNFR1 is associated with thoracic aortic calcification in patients with CKD stages 3 and 4 [67].

#### 3.1.2. Interleukin-1β (IL-1β)

IL-1β is a highly pro-inflammatory molecule, mainly produced by macrophages despite mRNA expression in many cell types, that stimulates the production of a broad spectrum of inflammatory cytokines, including IL-6. IL-1β is synthesized as a precursor, which must be cleaved by inflammasome-activated caspase-1 to be secreted in an active form. The biological activity of IL-1β depends on both IL-1β and the IL-1β receptor antagonist (IL-1ra), which competitively binds to the IL-1β receptor, thereby inhibiting IL-1β activity. In CKD, both IL-1β and IL-1ra levels are increased [58]. Therapeutics targeting the IL-1β pathway have been developed, notably, the IL-1ra anakinra and the canakinumab antibody. A randomized, placebo-controlled trial in hemodialysis patients showed that administration of IL-1ra reduced C-reactive protein (CRP) and IL-6, highlighting the contribution of IL-1β to inflammation in CKD [68]. Importantly, blocking the IL-1β pathway reduces cardiovascular events in CKD patients, as demonstrated by the CANTOS (Canakinumab Anti-inflammatory Thrombosis Outcomes Study) secondary prevention trial, which assessed the effect of IL-1β inhibition by canakinumab on the risk of major cardiovascular events in 1875 CKD patients [69].

#### 3.1.3. Interleukin-6 (IL-6)

IL-6 is a potent activator of the acute-phase response, which induces the hepatic production of CRP. IL-6 signaling in humans occurs via two main mechanisms: conventional IL-6 signaling, in which circulating IL-6 binds to transmembrane IL-6 receptor (IL-6R), and trans-signaling, in which circulating IL-6 binds soluble IL-6R and elicits biological effects via cell gp130. Only a few cell types express the IL-6R and respond to IL-6 classic signaling, whereas gp130 is ubiquitously expressed throughout the human body. Therefore, IL-6 trans-signaling may mediate the pro-inflammatory effects of IL-6 in the cell types directly implicated in the pathophysiology of cardiovascular disease, like endothelial cells, whereas conventional IL-6 signaling does not occur in vascular endothelial cells lacking IL-6R, except in the brain [70]. In dialysis patients, IL-6 and soluble IL-6R levels are increased, suggesting greater IL-6 trans-signaling in these patients [58], and both predict mortality of incident patients on hemodialysis or peritoneal dialysis [71]. In addition, in a randomized controlled trial in patients with chronic coronary syndrome stratified according to the level of renal function, an elevated IL-6 level was associated with an increased risk of major cardiovascular events in all CKD strata (normal kidney function, mild CKD, and moderate to severe) [72]. Recently, inhibition of IL-6 with clazakizumab was shown to reduce inflammation in hemodialysis patients, paving the way for its potential use in reducing cardiovascular events in CKD [73].

#### 3.1.4. Interleukin-18 (IL-18)

IL-18 is a pro-inflammatory cytokine involved in atheromatous plaque formation [74] that can reinforce the atherogenic process by increasing the expression of other pro-inflammatory molecules. Indeed, an over-expression of IL-18 and its receptor are found in macrophages, T cells, endothelial cells, and VSMCs in atherosclerotic plaque [74]. In ApoE^−/−^ mice, the administration of IL-18 enhances atherosclerosis, whereas a deficiency of IL-18 signaling inhibits the process of atherosclerosis development and stability [74]. In VSMCs, IL-18 activates inflammatory signaling pathways including SRC kinase, protein kinase C, p38 and JNK MAPKs, Akt, and the transcription factors NF-κB and AP-1, and it upregulates the expression of the pro-inflammatory molecules MCP-1, IL-8, and IL-6 [75]. The effects of IL-18 on NF-κB activation and cytokine gene expression are amplified by angiotensin II, which increases the expression of the α chain of the IL-18 receptor via STAT-3 [75]. In hemodialysis patients, IL-18 plasma levels are two-fold higher before dialysis than in control subjects and are further increased at the end of the dialysis session [76]. IL-18 serum levels predict 2-year cardiovascular mortality in non-diabetic CKD patients with a history of acute myocardial infarction within the previous year [77]. Furthermore, plasma IL-18 levels are associated with coronary artery calcium content and thoracic aortic calcification in patients with CKD stages 3 and 4 [67].

#### 3.1.5. Interleukin-8 (IL-8)

IL-8 is a chemoattractant produced by macrophages and endothelial and epithelial cells involved in the recruitment of neutrophils to sites of infection or tissue injury [58], which promotes the proliferation and invasiveness of VSMCs [78,79]. Studies suggest that IL-8 participates in vascular calcifications as well as in the pathogenesis of intimal hyperplasia. Exposure of endothelial cells to uremic plasma increases IL-8 expression [80], which promotes calcification in rat aortic rings [81]. IL-8 blockage markedly inhibits the proliferative/migratory phenotype of VSMCs [78] and prevents the increase in arterial intimal thickening in a human organ culture model [82]. The plasma levels of IL-8 are elevated in patients with CKD even before the stage of hemodialysis [83,84] and are associated with coronary artery calcium content in patients with CKD stages 3 and 4 [67]. In addition, in a small population of HD patients (n = 76) followed for 18 months, IL-8 independently predicted all-cause and cardiovascular mortality, even after adjustment for age, dialytic age, diabetes, and body mass index [85].

### 3.2. Asymmetric Dimethylarginine (ADMA)

Asymmetric dimethylarginine (ADMA) and symmetric dimethylarginine (SDMA) are endogenous inhibitors of NO synthesis that competitively bind to NO synthase and promote its uncoupling, thereby increasing ROS production and further reducing cardiovascular NO bioavailability. In patients with CKD, plasma levels of ADMA and SDMA are associated with cardiovascular and all-cause mortality [86,87].

The increase in ADMA levels in CKD appears to be mainly due to a decrease in the activity of ADMA-metabolizing enzymes rather than a decrease in urinary ADMA excretion [1]. ADMA has low protein binding and is 20–40% cleared by dialysis [1]. In mice, long-term treatment with ADMA induces significant lesions in coronary microvessels but not in the large coronary arteries [88]. In the endothelium, ADMA increases the production of ROS [89] and induces a pro-inflammatory phenotype through the activation of MAPK and NF-κB pathways [89,90]. ADMA upregulates endothelial MCP-1 expression [23], enhances the synthesis of TNFα [89], upregulates IL-8 and its receptor CXCR2 [89], increases the synthesis of ICAM-1 by endothelial cells [90], and, consequently, promotes the binding of monocytes to endothelial cells [89]. ADMA may additionally contribute to atherogenesis by promoting foam cell formation [1]. ADMA interacts with the renin–angiotensin system (RAS) and likely participates in the inflammatory effects of angiotensin II, which increases ADMA synthesis by endothelial cells [89]. In turn, ADMA upregulates angiotensin-converting enzyme (ACE) and increases superoxide production through the angiotensin II receptor, AT1 [88].

As for SDMA, it inhibits the anti-inflammatory function of HDL cholesterol by transforming physiological HDL into an abnormal lipoprotein that induces endothelial dysfunction [91]. In CKD patients, the accumulation of SDMA in HDL particles increases endothelial oxidative stress, reduces NO bioavailability, and promotes an endothelial pro-adhesive phenotype through VCAM-1 upregulation, leading to greater leukocyte adhesion and augmented arterial blood pressure [91].

### 3.3. Homocysteine

Homocysteine is a nonessential sulfur-containing amino acid resulting from the catabolism of methionine or cystathionine whose blood levels are influenced by vitamins B6, B12, and folic acid. Patients with CKD have higher blood homocysteine levels, probably due to impaired homocysteine metabolism in the kidneys rather than a reduced glomerular filtration rate [92]. In blood, about 90% of homocysteine is protein bound [92].

In hemodialysis patients, homocysteine is independently associated with a high risk of vascular and all-cause mortality [23]. In animal models of hyperhomocysteinemia and in cultured endothelial cells, homocysteine reduces NO bioavailability by accelerating NO oxidative inactivation [23]. Aortic endothelium isolated from hyperhomocysteinemic rats and endothelial cells incubated with homocysteine display increased oxidative stress [93] and activation of the NF-κB pathway evidenced by IKK activation, enhanced NF-κB/DNA binding activity, and increased transcriptional activity of NF-κB [93]. Homocysteine increases the mRNA expression and release of MCP-1 and IL-8 in cultured human aortic endothelial cells, thus promoting chemotaxis of human peripheral blood monocytes [94].

### 3.4. Urea

Elevated urea levels in CKD can lead to the generation of guanidines and the carbamylation of proteins, which are thought to contribute to oxidative stress, endothelial dysfunction, and inflammation [95]. Urea concentration is associated with cardiovascular disease [96].

### 3.5. Advanced Glycation End Products (AGEs)

AGEs are a group of diverse compounds derived from the nonenzymatic glycation of amino acids, peptides, proteins, or lipids by carbohydrates and other glucidic metabolites. AGEs accumulate in patients with diabetes and/or CKD as a result of inflammation, oxidative stress, and carbamoylation. In turn, AGEs promote pro-inflammatory, procoagulant, and pro-oxidant responses in vascular cells primarily through activation of their membrane-bound AGE receptor (RAGE) [97].

AGEs stimulate the production of ROS through NAD(P)H oxidase activation [98] and affect endothelial NO generation by reducing the expression of endothelial NO synthase [99]. AGE/RAGE interaction considerably decreases the endothelial production of PGI2 [100], increases the expression of tissue factor in endothelial cells and macrophages through NADPH activation [98,101], and enhances the anti-fibrinolytic activity of endothelial cells via PAI-1 upregulation [100]. In addition, AGEs favor the synthesis of inflammatory cytokines IL-1β and TNFα by macrophages [98,101]. In the vessels of diabetic ApoE^−/−^ mice and in cultured endothelial cells, RAGE activates NF-κB and enhances the expression of VCAM-1, leading to increased numbers of inflammatory cells adherent to the endothelium [98,101,102]. In 111 patients with stages 3b to 5 CKD, with a median follow-up time of 39 months, AGEs were independently associated with all-cause mortality [103].

### 3.6. Gut-Derived Uremic Toxins

#### 3.6.1. Trimethylamine N-Oxide (TMAO)

TMAO is produced in the liver by flavin-containing monooxygenase-3 (FMO3) from trimethylamine, a by-product of the metabolism of dietary choline, phosphatidylcholine, L-carnitine, and betaine by intestinal bacteria. As a water-soluble toxin, TMAO is efficiently cleared by dialysis.

Elevated TMAO levels are associated with inflammation, oxidative stress, and cardiovascular complications in CKD [86]. Studies have strongly suggested the role of TMAO in vascular calcifications and in atherosclerosis. In patients with CKD, serum levels of TMAO are higher in those with aortic arch calcification [104] and independently predict the coronary atherosclerosis burden and long-term mortality [105]. In rodents, TMAO increases macrophage recruitment to aortic lesions [1] and promotes the development of abdominal aortic aneurysms [106]. In vitro, TMAO increases endothelial permeability [1], induces monocyte adhesion to endothelial cells via the PKC/NF-κB/VCAM-1 pathway [107], and increases tissue factor expression [108]. TMAO exacerbates vascular calcifications induced by CKD in rodents and upregulates the osteogenic differentiation-related genes *RUNX2* and *BMP2*, leading to VSMC calcification in vitro [104]. The worsening of the atherosclerotic process and vascular calcifications by TMAO occurs through increased mitochondrial ROS production, NF-κB signaling, and NLRP3 inflammasome activation [1,104].

#### 3.6.2. Hippuric Acid

Hippuric acid is derived from dietary polyphenols converted by the gut microbiome into benzoic acid, which is subsequently conjugated with glycine in the liver or kidneys to form hippuric acid. Hippuric acid is 64% cleared by dialysis [1]. Hippuric acid may exert vascular pro-inflammatory effects through oxidative stress, as the treatment of cultured human aortic endothelial cells and rat aortic tissue with hippuric acid reduces eNOS expression while increasing ICAM-1 expression via the overproduction of mitochondrial ROS [109]. Hippuric acid is associated with left ventricular hypertrophy in dialysis patients [1] and with subclinical cardiac dysfunction in asymptomatic male patients with stages 2–5 CKD [110].

#### 3.6.3. p-Cresyl Sulfate

p-Cresyl sulfate is produced by the sulfation in the liver of p-cresol metabolized by intestinal bacteria from tyrosine and phenylalanine. Because of its high protein binding, p-cresyl sulfate is poorly cleared by dialysis [111]. Elevated serum p-cresyl sulfate levels are associated with the onset and progression of carotid atherosclerosis in hemodialysis patients [112]. In a cohort of 523 patients with stages 1–5 CKD followed prospectively, free p-cresyl sulfate was associated with the occurrence of fatal or non-fatal cardiovascular events in multivariate Cox regression models adjusted for age, sex, systolic blood pressure, diabetes mellitus, and estimated glomerular filtration rate [113]. The mechanism underlying the effects of p-cresyl sulfate may involve the induction of oxidative stress and vascular inflammation, leading to atherogenesis. In cultured human endothelial cells and in aortic VSMCs, p-cresyl sulfate increases ROS production and NADPH oxidase expression [114]. p-Cresyl sulfate increases the expression of the pro-inflammatory factors MCP-1 and TNFα in endothelial cells and monocytes [112], and it upregulates the expression of the endothelial adhesion molecules E-selectin, ICAM-1, and VCAM-1 [112], promoting leukocyte adhesion to the endothelium in vitro and in vivo and accelerating atherogenesis in ApoE^−/−^ CKD mice [112].

p-Cresyl sulfate activates the prostaglandin and inflammasome pathways in VSMCs by increasing the expression of cPLA2/COX-2, caspase-1, IL-1β, and NLRP3 inflammasome in an ROS-dependent way [20]. p-Cresyl sulfate may also be involved in vascular calcifications by inducing the expression of osteoblast-specific proteins in VSMCs via intracellular ROS generation and activation of the ERK/JNK/P38 MAPK and NF-κB pathways [20,114].

#### 3.6.4. Tryptophan-Derived Uremic Toxins

Tryptophan metabolism includes three major pathways in the gut intestinal tract: the kynurenine pathway in both epithelial and immune cells; the serotonin production pathway in enterochromaffin cells; and the indolic pathway, leading to the transformation of tryptophan into indole and its derivates by the intestinal microbiota [115].

##### Uremic Toxins from the Kynurenine Pathway

Ninety-five percent of tryptophan is metabolized into kynurenine by tryptophan 2,3-dioxygenase (TDO) and the rate-limiting enzymes indoleamine-2,3-dioxygenases (IDOs). TDO is highly expressed in the liver, while IDO exists in various organs such as the liver, brain, and intestinal tract [116]. After its production, kynurenine is metabolized through two distinct pathways into several metabolites, such as kynurenic acid and quinolinic acid [117]. Plasma levels of kynurenine and its metabolites, including kynurenic acid, 3-hydroxykynurenine, and quinolinic acid, are elevated in CKD patients on hemodialysis [118]. This accumulation is due not only to dysregulation of tryptophan metabolism during CKD, but also to the inability of hemodialysis to fully remove these toxins because of their binding to albumin. In addition, CKD patients have high levels of IDO and kynurenine and low levels of tryptophan, and the kynurenine/tryptophan ratio correlates with the stages of CKD [119].

Chronic inflammation in CKD patients may induce activation of the kynurenine pathway. Studies suggest that pro-inflammatory molecules such as TNFα, IL-6, and INFγ enhance IDO activity, which increases the degradation of tryptophan into the kynurenine pathway [120]. In turn, uremic toxins from the kynurenine pathway may induce inflammation, endothelial dysfunction, and oxidative stress. This is supported by studies in CKD patients, which demonstrate that the levels of kynurenine, kynurenic acid, and quinolinic acid increase with CKD severity and are positively correlated with hsCRP and TNFR1 [121]. 3-hydroxyanthranilic acid is significantly associated with MCP-1 levels [122], and the kynurenine/tryptophan ratio correlates with hsCRP in hemodialysis patients [123]. Additionally, the role of kynurenines in endothelial dysfunction was highlighted in a cross-sectional study in CKD patients showing that kynurenine pathway metabolites are associated with the endothelial markers von Willebrand factor, thrombomodulin, sICAM-1, and sVCAM-1 [124]. Finally, the association between kynurenines and oxidative stress was demonstrated in CKD patients [125] and confirmed in an animal study showing that kynurenine increases ROS production in endothelial cells and VSMCs independently of NADPH oxidase [126]. This oxidative stress decreases NO bioavailability and leads to a diminished NO-mediated vasorelaxation response of the rat aorta [126].

Free kynurenine concentration in serum is associated with cardiovascular mortality in patients with CKD stages 3 and 4 [127]. Additionally, studies show an association between toxins from the kynurenine pathway and atherosclerotic parameters in CKD patients. Kynurenine, 3-hydroxykynurenine, quinolinic acid, and the kynurenine/tryptophan ratio are associated with artery intima-media thickness [123,124]; 3-hydroxykynurenine levels are independently linked to the presence of CVD [125]; and the kynurenine/tryptophan ratio is associated with larger carotid plaques and decreased ankle-brachial pressure index in hemodialysis patients [123].

##### Uremic Toxins from the Indolic Pathway

The indolic pathway of tryptophan metabolism starts with the degradation of ingested tryptophan by the gut microbiota into indole through the activation of tryptophanase, which is found in many bacterial species such as *E. coli*, *Clostridium* spp., and *Bacteroides* spp. According to the EUTOX database (https://database.uremic-toxins.org/home.php (accessed on 21 November 2024)), uremic toxins from the indolic pathway include indole-3-acetic acid (IAA), indoxyl-β-D-glucuronide, and indoxyl sulfate. IAA is metabolized directly in the intestine or in tissues via tryptamine, whereas indoxyl sulfate and indoxyl-β-D-glucuronide are metabolized from indole in the liver [128]. Indolic uremic toxins are protein-bound molecules that are badly removed by hemodialysis or hemodiafiltration therapies [111].

Several studies strongly suggest the involvement of indolic uremic toxins in cardiovascular complications of CKD. Indoxyl sulfate levels are related to aortic calcification and vascular stiffness [129], and they predict overall and cardiovascular mortality in CKD patients, even after adjustment for age, gender, diabetes, albumin, hemoglobin, phosphate, and aortic calcification [129]. CKD patients with high serum levels of IAA experience more cardiovascular events and mortality than those with low levels [130], and serum IAA remains a significant predictor of these events independently of demographic and cardiovascular risk factors [130].

The cardiovascular toxicity of indolic uremic toxins may be mediated by the induction of a pro-oxidant and pro-inflammatory state associated with endothelial and VSMC dysfunction. In CKD patients, IAA levels correlate with the oxidative stress marker malondialdehyde [130], and serum levels of indolic toxins are positively associated with the levels of inflammatory markers such as IL-6, TNFα, CRP, and MCP-1 [130,131,132,133], as well as the endothelial adhesion molecules ICAM-1 and VCAM-1 [134], both before and after starting dialysis treatment. In vitro, endothelial ROS production is enhanced by treatment with IAA and indoxyl sulfate, the latter inducing an increase in NADPH oxidase activity and a decrease in glutathione levels [130,135]. Indolic uremic toxins induce a pro-inflammatory phenotype in vascular cells related to the activation of their receptor, the aryl hydrocarbon receptor (AHR, further developed), and of several inflammatory signaling pathways, including p38 and ERK1/2 MAPK, NF-κB, and AP-1 [130,136,137,138,139]. Indoxyl sulfate treatment increases IL-6 expression in rat aortic tissue, in cultured endothelial cells, and in VSMCs through OAT3/AHR/NF-κB pathway activation [138]. Indolic toxins activate the prostaglandin pathway by inducing an upregulation of COX-2 at both the mRNA and protein levels through the activation of tyrosine kinases and the AHR/p38MAPK/NF-κB pathway [130,140], leading to increased PGE_2_ secretion in cultured arterial and venous endothelial cells [130,140,141,142]. COX-2 inhibition and the subsequent blockage of PGE_2_ secretion strongly attenuated indoxyl sulfate-induced endothelial cell apoptosis [141], suggesting that activation of the COX-2/PGE_2_ axis may mediate indoxyl sulfate toxicity toward endothelial cells. Additionally, indoxyl sulfate promotes the synthesis of endothelial MCP-1 chemokine via AHR and ROS-mediated NF-κB activation [137,143] and upregulates the expression of the endothelial adhesion molecule ICAM-1, through the ROS-mediated activation of NF-κB [137]. Under inflammatory conditions, i.e., TNFα stimulation, indoxyl sulfate increased the expression of E-selectin, presumably through the AHR, JNK/AP-1, and NF-κB pathways [139,144]. Consequently, indoxyl sulfate dramatically enhances TNFα-induced leukocyte adhesion to cultured endothelial cells under physiological flow conditions and promotes leukocyte recruitment to the vascular wall in mice [139,144].

Indolic toxins promote a pro-oxidant, pro-inflammatory, and pro-calcifying phenotype of VSMCs leading to VSMC proliferation, migration, and calcification. Indoxyl sulfate stimulates VSMC proliferation and migration through ROS production, MAPK activation, and CREB/ATF3 signaling [145,146,147]. Indoxyl sulfate-mediated ROS production induces EGFR expression [148], which enhances angiotensin II signaling and increases VSMC migration [148]. Indoxyl sulfate promotes VSMC osteoblastic differentiation through JNK-related Pit-1 overexpression, leading to VSMC calcification [149]. Indoxyl sulfate may also enhance VSMC proliferation and calcification indirectly through cross-talk between endothelial cells and VSMCs. It increases the production of endothelial microvesicles [150], which induce TGF-β synthesis in VSMCs and activation of the Akt, ERK1/2, p38 MAPK, and Smad3 signaling pathways downstream of TGF-β, leading to enhanced VSMC proliferation [151]. Endothelial microvesicles from indoxyl sulfate-treated endothelial cells also modify the expression of pro-inflammatory genes coding for TNFα, IL-6, MCP-1, and RANTES and the pro-calcifying genes *RUNX2* and *BMP2* in VSMCs [152]. Furthermore, in cultured endothelial cells exposed to phosphate, indoxyl sulfate induces the expression and release of IL-8, which promotes VSMC calcification by preventing the induction of the powerful calcification inhibitor osteopontin [81].

Finally, indolic uremic toxins may promote thromboinflammation through the overexpression of tissue factor in arterial and venous endothelial cells as well as in endothelial microvesicles and VSMCs [136,142,153]. The upregulation of tissue factor in response to indolic toxins involves a transcriptional AHR/p38MAPK/NF-κB pathway in the endothelial cells [136] and an AHR-mediated inhibition of tissue factor ubiquitination, prolonging tissue factor half-life, in VSMCs [153,154].

**Figure 1:**
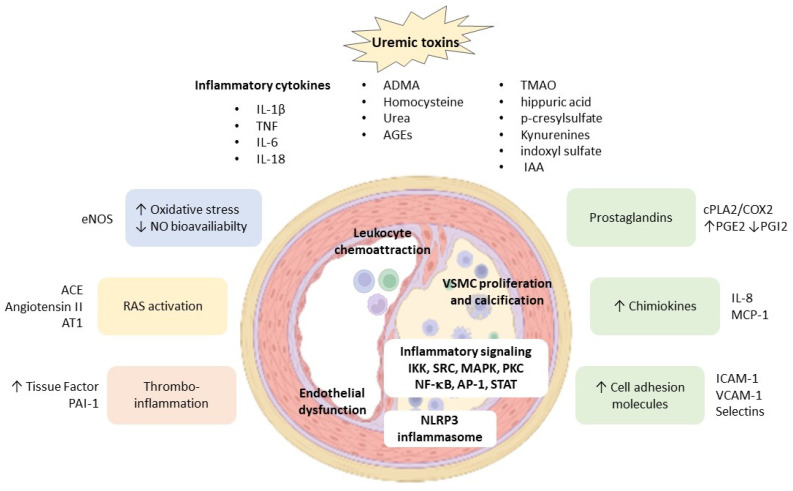
Vascular inflammatory mechanisms induced by uremic toxins.

An overview of the mechanisms involved in vascular inflammation induced by uremic toxins is provided in Figure 1.

## 4. AHR Activation: A New Mechanism in Vascular Inflammation Induced by Tryptophan-Derived Uremic Toxins?

Uremic toxins derived from tryptophan metabolism share the property of being AHR agonists, and their elevated levels in patients undoubtedly make CKD the chronic condition with the highest levels of AHR agonists. The AHR activation induced by these agonists plays a crucial role in vascular inflammation and its complications in CKD, as demonstrated in numerous studies [128,155,156,157].

**Figure 2:**
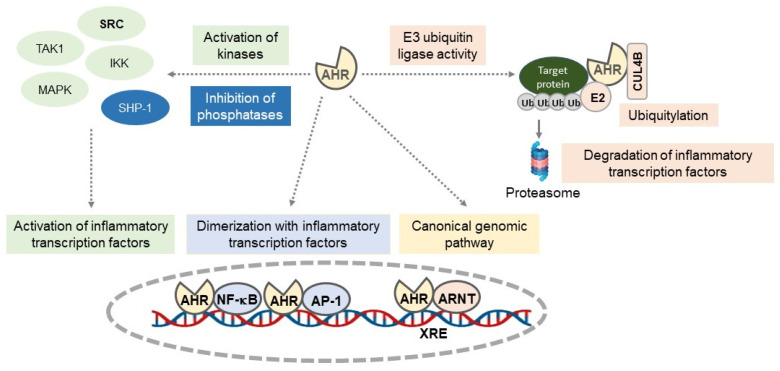
Regulation of inflammatory pathways by the aryl hydrocarbon receptor (AHR). AHR regulates inflammatory pathways through several mechanisms: by binding the AHR–ARNT complex to XREs in the regulatory regions of inflammatory genes (canonical genomic pathway); by interacting with subunits of other inflammatory transcription factors such as NF-κB or AP-1; by modulating kinase (SRC, TAK1, MAPK, IKK) or phosphatase (SHP-1) activities; and through its E3 ubiquitin ligase function, where AHR regulates the protein expression of inflammatory transcription factors or inflammatory proteins. AHR: aryl hydrocarbon receptor; ARNT: aryl hydrocarbon receptor nuclear translocator; TAK1: transforming growth factor-β (TGF-β)-activated kinase 1; IKK: IκB kinase; MAPK: mitogen-activated protein kinase; SHP-1: SRC homology region 2 domain-containing phosphatase-1/Tyrosine-protein phosphatase non-receptor type 6; NF-κB: nuclear factor-κB; AP-1: Activator Protein-1; XRE: Xenobiotic Response Element.

AHR is a member of the PER-ARNT-SIM superfamily of transcription factors, in which the PER-ARNT-SIM domain detects both endogenous factors, such as uremic toxins from dietary tryptophan metabolism, and exogenous factors, such as polyaromatic hydrocarbons or 2,3,7,8-tetrachlorodibenzo-p-dioxin (TCDD), its canonical agonist. AHR is contained in a protein complex composed of a 90 kDa dimer of Heat Shock Protein (HSP90), the AIP (or XAP2) protein, the co-chaperone p23, and the protein kinase SRC. This complex maintains AHR in the cytoplasm in a high-affinity conformation for its ligands and prevents its degradation by the proteasome [158]. The binding of an agonist to AHR leads to the transport of the AHR–ligand complex to the nucleus, where AHR binds to the aryl hydrocarbon receptor nuclear translocator (ARNT). In the nucleus, the AHR–ARNT complex is recruited to Xenobiotic Response Elements (XREs) in the regulatory regions of AHR target genes like CYP1A1 and regulates gene expression via the canonical genomic pathway [158]. AHR can also physically interact with the subunits of other transcription factors like NF-κB, for which physical interactions of AHR with RelA (p65), RelB, or other members of the NF-κB signaling complex have been demonstrated [158,159,160]. The binding of the AHR/RelA or the AHR/RelB complexes to the NF-κB response elements on IL-6 and IL-8 promoters increases the expression of these genes [159,160]. In turn, the activation of NF-κB signaling by inflammatory stimuli increases the expression of AHR and its repressor AHRR, whose promoters contain functionally active NF-κB binding sites [161,162]. An enhanced AHR expression mediated by inflammation would lead to increased sensitivity toward AHR ligands and the modulation of AHR-dependent gene expression [161]. AHR may also regulate inflammatory transcription factors through its E3 ubiquitin ligase function, which induces the ubiquitination and degradation of target proteins by the proteasome, as demonstrated for the FOS subunit of the transcription factor AP-1 [158], or for NF-κB RelA/p65 in macrophages [163]. Finally, AHR may participate in inflammatory signaling via phosphorylation cascades initiated by the release of SRC kinase from the ligand-activated, AHR-triggered chaperone complex or by the interaction of AHR with other kinases like TAK1 or MKK3/6 [158,164,165]. AHR may also regulate the activity of phosphatases, as demonstrated in monocytes, where its activation modulates the activity of STAT6 by reducing the activity of its inhibitory phosphatase, SHP-1 [166]. Taken together, these data show complex cross-talk between AHR and inflammatory pathways (Figure 2), with AHR being either pro- or anti-inflammatory, depending on the context, cell type, and AHR agonist.

**Figure 3:**
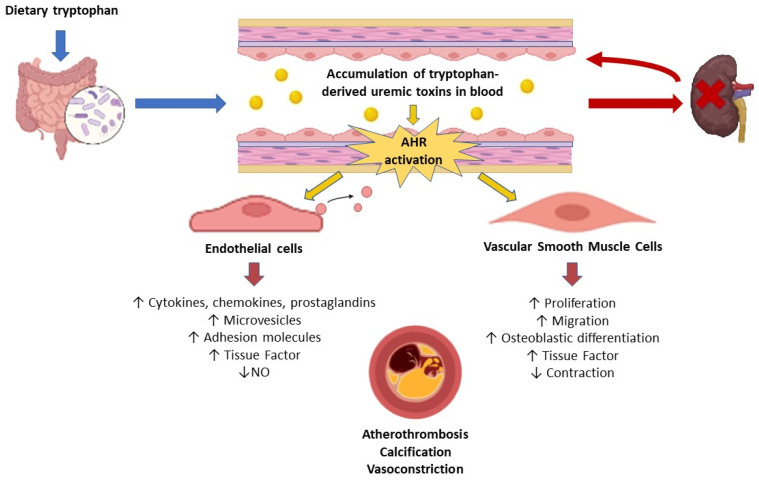
Mechanisms induced by tryptophan-derived uremic toxins leading to vascular dysfunction. Tryptophan-derived uremic toxins are produced in the gut via the metabolism of dietary tryptophan. Their accumulation in the blood resulting from impaired renal elimination induces endothelial and smooth muscle cell dysfunction through the activation of the aryl hydrocarbon receptor (AHR). The impairment of endothelial and smooth muscle cell function and phenotype contributes to atherothrombosis, vascular calcification, and vasoconstriction.

The activation of the AHR pathway in CKD has been clearly demonstrated in humans and mice [155]. CKD patients showed increased expression of the AHR target genes *CYP1A1* and *AHRR* in blood cells [155]. The AHR-activating potential (AHR-AP) of the CKD patients’ serum, reflecting the serum’s ability to activate AHR ex vivo, was increased compared with the control subjects and correlated with the glomerular filtration rate and serum levels of indoxyl sulfate [155]. Mice with CKD and mice injected with indoxyl sulfate display AHR activation in the cardiovascular system, evidenced by an increased expression of the AHR target gene *Cyp1a1* in the aorta and heart, which is associated with an elevation of the serum AHR-AP [155]. Cardiovascular events are more frequent in CKD patients with elevated AHR-AP, suggesting that AHR activation is one of the mechanisms involved in cardiovascular complications of CKD [155]. Experiments have shown the pro-inflammatory effect of AHR activation by indolic uremic toxins in vascular cells. Endothelial cells incubated in vitro with indolic toxins display a strong overexpression of genes regulated by AHR [142], and the pro-inflammatory phenotype induced by indolic toxins appears to depend on the signaling pathways activated by AHR [130,136,140,142,143]. Indeed, AHR controls the endothelial overexpression of tissue factor and COX-2 via a non-genomic pathway in which AHR activates p38 signaling that leads to NF-κB activation [130,136]. In addition, AHR appears to be upstream of the activation of the NF-κB and JNK/AP-1 pathways that mediate E-selectin upregulation by indoxyl sulfate in endothelial cells [139]. AHR is involved in the amplification of TNF-mediated leukocyte adhesion by indoxyl sulfate, as this leukocyte adhesion is significantly reduced by silencing AHR in endothelial cells and in endothelial cell-specific AHR KO mice [139]. AHR is also responsible for indoxyl sulfate-induced NF-κB p65 signaling transduction, which increases IL-6 expression in endothelial cells and VSMCs [138]. Furthermore, the activation of the AHR pathway by indoxyl sulfate in aortic VSMCs prevents AHR-related tissue factor ubiquitination and degradation by the proteasome, leading to the increased protein expression of tissue factor [154]. Finally, AHR inhibition ameliorates the increase in oxidative stress, leading to a decreased NO-mediated vasorelaxation response induced by kynurenine treatment of the endothelium-intact aorta, but not of the endothelium-denuded aorta, supporting the idea that AHR is involved in oxidative stress in endothelial cells but not in VSMCs [126]. The role of the tryptophan-derived uremic toxins/AHR axis in vascular dysfunction is illustrated in Figure 3.

Targeting AHR could be a promising therapy for limiting vascular inflammation in CKD, keeping in mind that its anti-inflammatory effects may depend on the inhibitor/agonist/antagonist used and the cell type [167,168]. The dietary AHR agonist indole-3-carbinol and its intestinal metabolite 3,3′-diindolylmethane have anti-inflammatory effects through reduced ROS production in macrophages and decreased phosphorylation of IκBα, leading to inhibition of the NF-κB pathway [167]. Additionally, the AHR ligand polyphenols reduce NF-κB activation and the expression of numerous inflammatory mediators such as IL-8, TNFα, IL-6, and COX-2 in different cell types [167]. In patients with CKD on hemodialysis, a 3-month oral supplementation with the AHR ligand curcumin displays anti-inflammatory effects by significantly decreasing plasma levels of hsCRP and NF-κB expression in leukocytes [169]. On the other hand, in animal models of autoimmune or inflammatory diseases, AHR inhibition by CH223191, a pharmacologic inhibitor, is pro-inflammatory and pro-fibrosing [168]. In the context of cardiometabolic diseases, studies show that inhibiting AHR has rather beneficial effects on the cardiovascular system [168]. In mouse models of ischemic stroke, intraperitoneal administration of CH223191 prevents the overexpression of AHR and CYP1A1 in the cerebral cortex and striatum; decreases neuroinflammation through the suppression of TNF-ɑ, IL-1β, and COX-2 upregulation; and reduces infarct size and the severity of neurological damage [168]. More studies are needed to provide data on the beneficial effects of targeting AHR on vascular inflammation in patients with CKD. We must keep in mind that while AHR activation is clearly deleterious in the vascular wall in CKD, some preclinical data suggest a beneficial effect of AHR activation in the endothelium of the gut [170] and the lungs [171]. Physiological activation of AHR in these barrier organs could play a role in maintaining endothelial quiescence, thereby limiting inflammation and its harmful consequences. A better understanding of AHR pathway regulation is crucial for effectively targeting it to improve the health of patients with CKD while avoiding adverse side effects [172].

## 5. Conclusions

Inflammation plays a significant role in the progression of CKD, which in turn increases the inflammatory load, exacerbating renal and cardiovascular damage and raising the risk of cardiovascular disease. Uremic toxins, particularly those derived from tryptophan metabolism, are key contributors to vascular inflammation that can lead to cardiovascular disease. Targeting their receptor, AHR, may offer a novel strategy to mitigate vascular inflammation and reduce the cardiovascular burden in CKD.
